# A Review of the Combination of Experimental Measurements and Fibril-Reinforced Modeling for Investigation of Articular Cartilage and Chondrocyte Response to Loading

**DOI:** 10.1155/2013/326150

**Published:** 2013-04-08

**Authors:** Petro Julkunen, Wouter Wilson, Hanna Isaksson, Jukka S. Jurvelin, Walter Herzog, Rami K. Korhonen

**Affiliations:** ^1^Department of Clinical Neurophysiology, Kuopio University Hospital, FI-70211 Kuopio, Finland; ^2^Department of Biomedical Engineering, Eindhoven University of Technology, 5600 MB, Eindhoven, The Netherlands; ^3^Department of Applied Physics, University of Eastern Finland, FI-70211 Kuopio, Finland; ^4^Division of Solid Mechanics, Department of Orthopaedics, Lund University, 221 00 Lund, Sweden; ^5^Human Performance Laboratory, Faculty of Kinesiology, University of Calgary, Alberta, BC, Calgary, Canada T2N 1N4

## Abstract

The function of articular cartilage depends on its structure and composition, sensitively impaired in disease (e.g. osteoarthritis, OA). Responses of chondrocytes to tissue loading are modulated by the structure. Altered cell responses as an effect of OA may regulate cartilage mechanotransduction and cell biosynthesis. To be able to evaluate cell responses and factors affecting the onset and progression of OA, local tissue and cell stresses and strains in cartilage need to be characterized. This is extremely challenging with the presently available experimental techniques and therefore computational modeling is required. Modern models of articular cartilage are inhomogeneous and anisotropic, and they include many aspects of the real tissue structure and composition. In this paper, we provide an overview of the computational applications that have been developed for modeling the mechanics of articular cartilage at the tissue and cellular level. We concentrate on the use of fibril-reinforced models of cartilage. Furthermore, we introduce practical considerations for modeling applications, including also experimental tests that can be combined with the modeling approach. At the end, we discuss the prospects for patient-specific models when aiming to use finite element modeling analysis and evaluation of articular cartilage function, cellular responses, failure points, OA progression, and rehabilitation.

## 1. Introduction 

**Figure 1:**
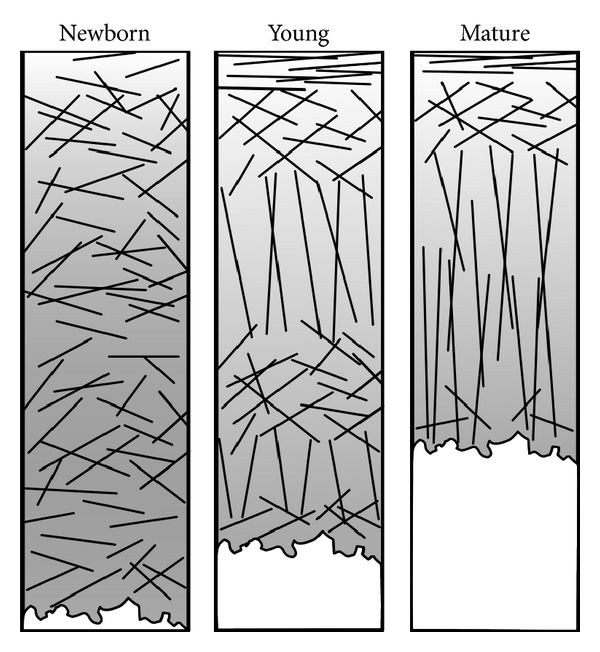
Presentation of the collagen network organization in maturing articular cartilage based on articular cartilage from rabbit, pig, and sheep [1–4]. When maturing, a nonorganized collagen fibril network slowly forms into a traditional Benninghoff-type arcade structure. At the same time, cartilage thickness is decreased [1, 2, 4, 15].

As a fluid-saturated composite tissue, articular cartilage provides smooth sliding at joint surfaces. Articular cartilage also absorbs forces and reduces stresses experienced by bones. The extracellular matrix (ECM) of cartilage can be divided in two phases: solid and fluid. The solid phase is primarily composed of cartilage matrix proteins: proteoglycans (PGs) and collagen (mainly type II). Cartilage also contains chondrocytes, that is, articular cartilage cells, which are surrounded by a thin layer called the pericellular matrix (PCM). The characteristic fibrous structure of the ECM is a result of depth-wise remodeling of the collagen fibril network during maturation [1–4] and represents a three-layer laminar architecture in adults (Figure 1) [5]. The collagen network is known to be arranged into parallel planes, revealing split-line patterns in cartilage surfaces [5]. The well-organized collagen fibril bundles link to each other with smaller cross-link chains. 

The mechanical response of articular cartilage is determined by the tissue composition and structure [6–10]. Due to the fixed negative charges of the PGs, the total ion concentration inside the tissue is higher than in the surrounding synovial fluid (cation concentration is higher and the anion concentration is lower), which leads to a difference in osmotic pressure that results in tissue swelling [11]. The swelling pressure modulates the shape and volume of the tissue by controlling the fluid content of the tissue [12]. The collagen network of the ECM stabilizes the matrix by balancing the expansion caused by the swelling pressure [13, 14]. 

The collagen network is believed to mainly affect the response of cartilage under dynamic or instantaneous loading [6, 7, 19–22], while its direct impact on the equilibrium response is minor [6, 7, 19, 21]. Oloyede and Broom utilized a consolidation theory and experiments to understand the role of interstitial fluid flow for the mechanical response and deformation behavior of loaded cartilage tissue [23–25]. During loading the interstitial fluid flows within the cartilage solid matrix. The flow velocity is determined by the induced fluid pressure and matrix permeability. Since the fluid flow is restricted by the cartilage matrix during mechanical loading, the internal pressure of the tissue increases. Therefore, the interstitial fluid also directly affects the dynamic and instantaneous mechanical responses of cartilage. 

Chondrocytes produce the ECM matrix components. The activity of chondrocytes is affected by genetic and environmental factors, electrokinetic forces, mechanical forces, and fluid pressurization. Chondrocytes change shape and volume in response to mechanical loading of cartilage [26–28]. The ECM and PCM possess organized collagenous structures, and the collagen fibril orientation is affected by local tissue strains [10], which further modulate cell shape and volume [27]. Consequently, these factors modulate the PCM and ECM composition [29–31]. Through a sequence of mechanobiological events, the mechanical signals from the ECM are converted into intracellular responses, which serve to maintain or alter cartilage ECM. The PCM around the cells acts as a transducer for the biomechanical and biophysical signals perceived by the cells and thus alters the cell activity [30].

Osteoarthritis (OA) is a joint disease, characterized by the progressive degeneration of articular cartilage. PG depletion occurs in the superficial cartilage during the early stage of OA [32–35]. Simultaneous alterations in the collagen network also take place [32, 35–37]. These alterations include a dramatic change in orientation of the superficial collagen fibrils [32, 34, 38, 39] and a reduction in the collagen content [34]. Thereby, these changes increase cartilage hydration via intake of water. Subsequently, tissue permeability increases. Together, these changes impair the mechanical function of cartilage by decreasing its mechanical stiffness [32], which may further accelerate the progression of OA. As a result of the OA changes in tissue composition and structure, the extracellular osmolarity increases. This results in increased cell volume, which may in turn affect tissue biosynthesis and cartilage mechanotransduction.

Changes in the cartilage structure and composition can be revealed by using *in vivo* imaging techniques, such as magnetic resonance imaging (MRI) or contrast-enhanced computed tomography (CECT) or *in vitro* techniques such as quantitative microscopy [2, 34, 40–46]. However, these imaging techniques alone are unable to quantify mechanical characteristics of cartilage that are critical to the demanding mechanical function in the joint. Instead, computational modeling in conjunction with imaging techniques is needed to predict the mechanical performance of cartilage and other tissues during joint loading [17, 32, 47–53].

Chondrocyte responses to osmotic or mechanical loading have been experimentally characterized by using confocal or dual-photon microscopy [54–56]. In earlier studies, both mechanical responses, for example, volume and morphology changes, and biological responses, such as Ca^2+^ signaling, have been characterized [54–57]. The measurements have traditionally been conducted on isolated cells or chondrocytes have been measured through the cut surface of tissue explants. Recently, cell responses to loading have also been conducted *in situ* using fully intact tissue samples, providing more physiological environment for cells [55, 56]. Like at the tissue level, the stresses, strains, and fluid flow in and around the cells cannot be measured experimentally. It is also experimentally challenging, if not impossible, to specifically investigate the roles of different cartilage constituents (e.g., collagen, PGs, fluid, and ions) on the cell responses. Therefore, computational models have been employed [58, 59].

The modern fibril reinforced computational models of articular cartilage may be implemented to include inhomogeneous tissue composition and complex structure [8, 32, 53, 59–68]. These models can simulate nonlinear behavior of the tissues, caused by tissue anisotropy and inhomogeneity. Cartilage models have been employed to evaluate the mechanical behavior of cartilage at the cell, tissue, and joint level, to evaluate static and dynamic tissue behavior, to explore the effects of mechanical and biochemical loading, and to predict tissue remodeling, growth, and adaptation over time. As a next step, the 3D models may be developed and validated to include patient-specific tissue characteristics. Ultimately, they may then become clinical tools for predicting the progression of OA and thus for identifying or optimizing patient-specific treatment strategies. To achieve this goal, a smooth transition is needed from joint-level models to cell-level imaging and modeling. This could help to characterize the effects of joint loads on tissues, and cells, to investigate possible failure sites in tissues, and to predict cell-based tissue adaptation to loading in normal and diseased joints. In this paper, we concentrate on cell- and tissue-level models and point out their future possibilities. Theories of present fibril reinforced models are reviewed, and major challenges in validation and application of these models are critically discussed. 

## 2. Review of Fibril Reinforced Computational Models of Articular Cartilage

This paper focuses on the application of fibril reinforced mixture models. The application of fibril reinforcement in the models is justified by the heavily influential collagen fibril network for the cartilage mechanical behavior [69, 70]. Oloyede and Broom considered that there are six aspects influencing the deformation behavior of cartilage which a theoretical or physical model should incorporate [71]: (1) the rate of fluid outflow from the tissue matrix, (2) the diffusion process or movement of interstitial fluid between adjacent regions of the matrix, (3) deformation dependent permeability, (4) loading-rate-dependent viscous drag, (5) physico-chemistry and osmotic swelling, and (6) the nonlinearity of the stress-strain characteristic of the solid skeleton or structural framework.

It is known that articular cartilage exhibits viscoelastic behavior [72–77]. The viscoelastic behavior is often divided to flow-dependent and flow-independent behavior [74, 78]. Prior to the development of fibril reinforced models, the biphasic mixture theory (fluid and solid phase) was introduced for cartilage by Mow et al. [78]. Use of the biphasic (and poroelastic) material models can at least partly account for the flow-dependent viscoelastic behavior, which arises from fluid flow within a porous material with low permeability [75, 76, 79–84]. The biphasic mixture theory has been demonstrated to agree well with direct experimental measures of fluid pressurization [23, 24, 83, 85]. While the theory was considered successful in many aspects, it failed to predict certain mechanical behavior observed under mechanical tests [86, 87]. The models were improved by transversely isotropic elastic [88] and conewise linear elastic [85] implementation of the solid matrix. Use of biphasic transversely isotropic models produced better curve fits as compared to biphasic linear elastic models [88, 89]. The transversely isotropic models were able to capture one aspect of the compression-tension nonlinearity of a cartilage having different mechanical properties in the direction of compression and perpendicular to that direction. However, those models could not account for the experimentally observed compression-tension nonlinearity, caused by the collagen fibrils, measured between compressive and tensile tests [21, 90, 91]. The conewise linear elastic model overcame this issue via a bimodular approach setting the material properties in the model different during compressive and tensile strain [85]. Soulhat et al. [92] implemented a composite fibril reinforced biphasic model in which the collagen fibrils were represented as springs over the solid matrix, resisting deformation only in tension. These are evolutionary steps taken towards present models used to simulate the mechanical behavior of articular cartilage. Further development of modern fibril reinforced models from that introduced by Soulhat et al. is reviewed later in this section.

Since it has been difficult to separate the flow-independent from the flow-dependent viscoelasticity, it has been questioned whether cartilage possesses flow-independent viscoelastic properties at all [80, 81, 93]. Some experimental evidence supports the existence of flow-independent viscoelastic properties in cartilage [94–99], which can partly be attributed to the collagen fibrils and partly to a lesser extent to the nonfibrillar matrix or their interaction [97, 100–105]. For modeling cartilage as a biphasic viscoelastic material, Mak [106] combined the biphasic theory with a quasilinear viscoelastic theory for the solid matrix by Fung [107]. This allowed for the separation of flow-dependent and flow-independent viscoelasticity. The more recent fibril reinforced models developed for articular cartilage have often implemented the fluid-independent viscoelastic behavior of the nonfibrillar solid, viscoelastic fibrils, or both [8, 12, 18, 68, 81, 108].

Recent mechanical simulation studies including articular cartilage have often implemented a fibril reinforced material model. However, for more complex geometries, the implementation of fibril reinforced articular cartilage is often omitted. Mostly, these approaches are used when implementation of fibril reinforcement is considered unnecessary (justified simplification) for the aim of the study. The inclusion of fibril reinforcement may sometimes be unnecessary due to the role of articular cartilage for the simulated result. Excessive computational cost may provide reasoning for simplification of the cartilage material model. For instance, it is still customary to model articular cartilage as isotropic elastic material during complete joint simulations [109–113]. However, recent models of a joint have included fibril reinforcement when simulating joint mechanics with focus on cartilage [53, 114–117]. Adouni et al. found that use of a fibril reinforced model in a total knee joint model exhibited larger contact pressures at knee joint contact surfaces when comparing to a simplified isotropic material model [114]. Often, the use of simplified material implementation is justified due to the short-term type of simulation during which viscoelastic behavior does not play a great role and articular cartilage appears as an incompressible and elastic solid [87]. Simplifying the material behavior of articular cartilage as an incompressible, single-phase material is appropriate when rapid loading with short duration is investigated. The equivalence of the short-time biphasic and incompressible elastic responses has been demonstrated [118]. In the following, we present fibril reinforced mixture models more in detail.

### 2.1. Tissue-Level Fibril Reinforced Models

The fibril reinforced modeling of articular cartilage at the tissue level started in the late nineties. Then for the first time, a model included specifically both the collagen network and the poroelastic matrix (PGs and fluid) [119]. Since then, development of computational models of cartilage has advanced quickly to include complex material characteristics models with more realistic behavior (Table 1). The main features of the fibril reinforced models of cartilage tissue are presented below. 

The first fibril reinforced models were composed of the fibrillar part, representing the collagen fibrils, and the nonfibrillar part, mimicking the porous material (mainly PGs) filled with fluid [7, 119]. Total stress (**σ**_*t*_) in this material can be expressed as:

(1) $$\begin{array}{c} \sigma_{t}=\sigma_{nf}+\sigma_{\text{fibril}}-pI, \end{array}$$

where **σ**_*nf*_ is the nonfibrillar matrix stress, **σ**_fibril_ is the fibril network stress, *p* is the fluid pressure, and **I** is the unit tensor. 

#### 2.1.1. The Nonfibrillar Matrix

The nonfibrillar part was modeled as linear elastic material in the original models obeying Hooke's law, while at larger strains a hyperelastic, Neo-Hookean material description is preferable [17, 18]. The constitutive equation for the Neo-Hookean material is

(2) $$\begin{array}{c} \sigma_{nf}=\frac{2}{J}C_{10}\left(\bar{B}-\frac{1}{3}\mathrm{tr}\left(\bar{B}\right)I\right)+\frac{2}{D_{1}\left(J-1\right)I}, \end{array}$$

where *C*_10_ and *D*_1_ are temperature-dependent material parameters, $\bar{B}$ is Cauchy-Green deformation tensor, and *J* is the elastic volume ratio. The material parameters can be expressed as

(3) $$\begin{array}{c} C_{10}=\frac{G_{0}}{2},\;\;D_{1}=\frac{3\left(1-2\nu_{nf}\right)}{G_{0}\left(1+\nu_{nf}\right)}, \end{array}$$

where *G*_0_ is the initial shear modulus and *ν*_*nf*_ is Poisson's ratio of the nonfibrillar matrix. The shear modulus can be defined via Young's modulus of the nonfibrillar matrix (*E*_*nf*_) and *ν*_*nf*_:

(4) $$\begin{array}{c} G_{0}=\frac{E_{nf}}{2\left(1+\nu_{nf}\right)}. \end{array}$$

Fluid flow in the nonfibrillar matrix has been generally modeled with Darcy's law:

(5) $$\begin{array}{c} q=-k\nabla P, \end{array}$$

where *q* is the flow rate, *k* is the permeability, and ∇*P* is the pressure gradient across the region. The permeability *k* can be defined to be dependent on the porosity and void ratio, that is, ratio of fluid to solid fraction, for example, according to the following equation given by van der Voet [120]:

(6) $$\begin{array}{c} k=k_{0}{\left(\frac{1+e}{1+e_{0}}\right)}^{M}, \end{array}$$

where *k*_0_ is the initial permeability, *e*_0_ and *e* are initial and current void ratios, and *M* is a positive constant [18, 120]. To be consistent with experimental findings, the fluid fraction that is used to compute the void ratios has also been implemented in the models in a depth-dependent manner over the cartilage thickness [18, 61, 64, 121–123]. In the end, the required material parameters for the nonfibrillar matrix of cartilage are Young's modulus (*E*_*nf*_), Poisson's ratio (*ν*_*nf*_), and permeability (*k*_0_). To include the effect of fibril reinforcement in the permeability, Federico and Herzog implemented the anisotropy of permeability by considering the orientation of the collagen fibrils in any given location in the tissue in the undeformed state [124]. Few recent studies have also considered intrinsic anisotropy of permeability due to the collagen fibrils [67, 125, 126]. Imaging studies measuring diffusion of water within the cartilage matrix have observed anisotropy of diffusion, mainly affected by the general collagen fibril orientation [127–129]. In addition, the variation in excess pore pressure in the different directions parallel and perpendicular to the surface of cartilage provides experimental support for the implementation [130]. If we were to include the anisotropy of permeability in (6), the results would be

(7) $$\begin{array}{c} k_{\left|\right|}=k_{\left|\right|}^{0}{\left(\frac{1+e}{1+e_{0}}\right)}^{M_{\left|\right|}},\\ k_{⊥}=k_{⊥}^{0}{\left(\frac{1+e}{1+e_{0}}\right)}^{M_{⊥}}, \end{array}$$

where *k*_||_^0^ and *k*_||_ are the initial and current permeability in the direction of collagen fibrils, respectively, *k*_⊥_^0^ and *k*_⊥_ are the initial and current permeability across the collagen fibrils, and *M*_||_ and *M*_⊥_ are material parameters. Ratio of *k*_||_ and   *k*_⊥_ in any given point in the model would then depend on the orientation of the collagen fibrils.

#### 2.1.2. Collagen Fibril Network

We divide the fibril reinforcement models into three types: (1) transversely isotropic [69, 88, 134] or conewise linear elastic [85], (2) fibril reinforcement with membrane elements or springs [7, 119, 131], and (3) vector-based fibril reinforcement [8, 12, 18, 68, 108, 135]. These types of models have been applied to simulate articular cartilage mechanics during the past decade. We will mainly focus our paper on the vector-based fibril reinforcement. 

**Figure 2:**
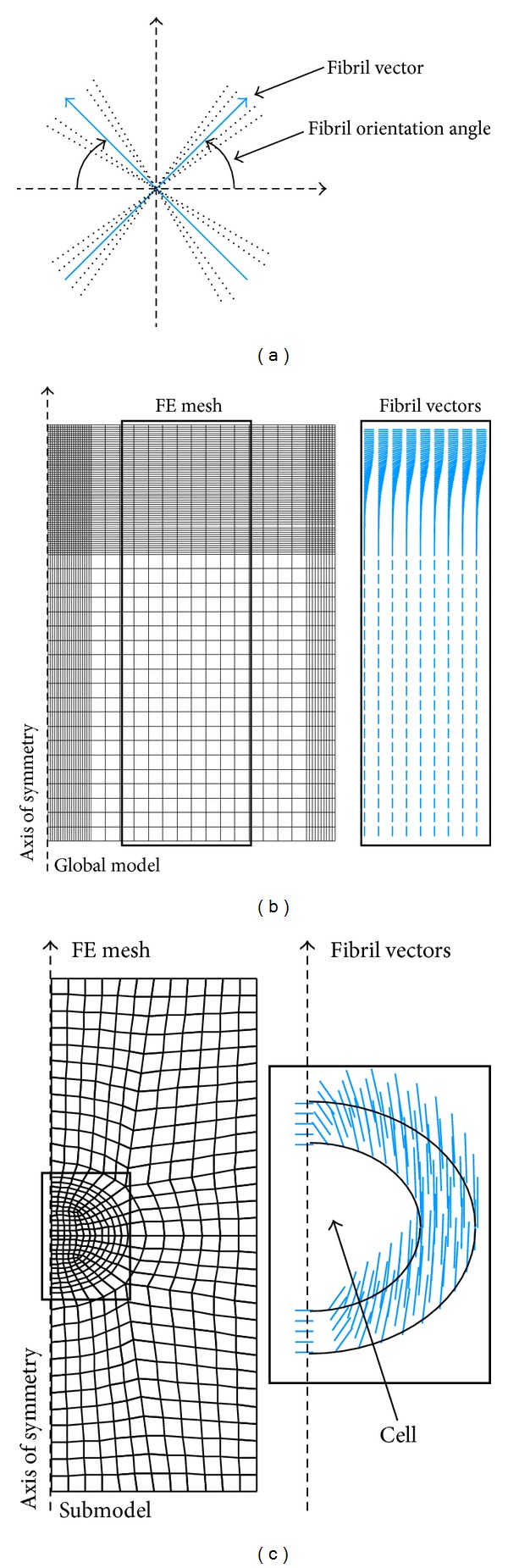
Fibril orientation in the vector-based fibril reinforced models can be implemented with any given structure. The principle is that the fibril vector is given a direction at each point in the model (a). Two examples of the implemented structure are presented [16]. On the left, (b) an articular cartilage sample is modeled in unconfined compression geometry with a typical Benninghoff-type arcade structure including the superficial, middle, and deep layer. On the right, (c) a submodel of the global model presented on the left is implemented with pericellular matrix fibrils tangential to the cell surface. The extracellular matrix fibril vectors are not presented in (c) for clarity. The axis of symmetry has been indicated.

In the early fibril reinforced models, the fibrillar matrix mimicked the mechanical effect of the collagen fibrils of cartilage [119]. The properties of the fibril network were controlled by Young's modulus of the fibril network (*E*_*f*_). The elastic properties of the fibril network were further characterized with a nonlinear relation as such relation has been reported in the literature [136]:

(8) $$\begin{array}{c} E_{f}=E_{f}^{0}+E_{f}^{\varepsilon }\varepsilon_{f},\;\text{for}\;\varepsilon_{f}>0, \end{array}$$

(9) $$\begin{array}{c} E_{f}=0,\;\text{for}\;\varepsilon_{f}\leq 0, \end{array}$$

where *E*_*f*_^0^ is the initial fibril network modulus, *E*_*f*_^*ε*^ is the strain-dependent fibril network modulus, and *ε*_*f*_ is the fibril strain [7, 119]. Fibrils were presented with spring elements [7, 119]. Another approach was to use nonlinear membrane elements [131]. As noticed from (8) and (9), the characteristic feature of the fibrils is that they resist only tensile forces. This is a major difference between transversely isotropic and spring-based models, the former having the same stiffness in compression and tension. Collagen fibrils have been demonstrated to possess flow-independent viscoelasticity [100–102]. Therefore, the collagen fibril stresses (*σ*_*f*_) were later modeled as viscoelastic:

(10) σf=η2(σf−Ef0εf)Efεσ˙f+Efεεf+(ηEf02(σf−Ef0εf)Efε+η)ε˙f, for  εf>0,σf=0, for  εf≤0,

where *η* is the viscoelastic damping coefficient, and $\dot{\sigma }$ and $\dot{\varepsilon }$ are the stress and strain rates, respectively [17, 18]. Unlike in type 1 and 2 models, the fibrils in the vector-based fibril reinforced models (type 3) can be constructed into any structural arrangement with fibril vectors (Figure 2) [18, 47, 53, 63, 68, 108]. This allows for the implementation of realistic fibril orientation to be independent of the mesh in use. The fibril vectors ($\vec{v}$) can be used for determining logarithmic fibril strain used in (10) [18]:

(11) $$\begin{array}{c} \varepsilon_{f}=\log||F\cdot \vec{v}||, \end{array}$$

where **F** is the deformation tensor.

The fibrillar matrix in the model can be divided into primary and secondary fibrils [18]. The primary fibrils may be assumed to represent the arcade-like collagen architecture, as detected with the polarized light microscopy [69, 137]. This network creates a depth-dependent tensile modulus for the tissue. As is commonly thought the fibrils are oriented parallel to the cartilage surface in the superficial zone, curve in the middle zone, and turn to perpendicular orientation with the cartilage surface in the deep zone [5]. The secondary fibrils mimic the less organized part of the collagen network which is observed in scanning electron microscopy [138]. When looking closely, the collagen fibrillar network appears random but exhibits highly directional configuration overall [37, 139]. The stresses for the primary and secondary fibrils (*σ*_*f*,*p*_ and *σ*_*f*,*s*_, resp.) can be formulated as follows:

(12) $$\begin{array}{c} \sigma_{f,p}=\rho_{z}C\sigma_{f}, \end{array}$$

(13) $$\begin{array}{c} \sigma_{f,s}=\rho_{z}\sigma_{f}, \end{array}$$

where *ρ*_*z*_ represents the fibril volume fraction and *C* is the density ratio of primary and secondary fibrils. The stress of the fibril network (**σ**_fibrils_) is finally determined as the sum of the stresses in each individual fibril (*σ*_*f*,all_^*i*^):

(14) $$\begin{array}{c} \sigma_{\text{fibrils}}=\sum_{i=1}^{\text{tot}f}\sigma_{f,\text{all}}^{i}, \end{array}$$

where tot*f* is the total number of fibrils used to implement the fibrillar structure in an integration point.

#### 2.1.3. Extensions to Fibrillar and Nonfibrillar Matrices

Tissue swelling is an essential part of cartilage mechanical behavior. Swelling properties of the fixed charge density in PGs have been presented in previous swelling cartilage models [6, 8, 12, 16, 59, 60, 64, 132, 140, 141]. Swelling of cartilage is influenced by osmotic swelling due to an excess amount of ion particles inside the tissue and chemical expansion due to repulsion of closely spaced negatively charged PGs [141, 142]. Lai et al. [143] introduced a triphasic mixture model, which includes three phases: an incompressible solid, an incompressible fluid, and a monovalent ionic phase. In the triphasic model, swelling behavior is explained by three mechanisms: Donnan osmosis, excluded volume effect, and chemical expansion [144]. Simon et al. [145] introduced an alternative model by including the swelling effects in fluid equilibrium. The fundamental difference between these two approaches is that the model by Lai et al. causes fluid flow within the tissue, while the model by Simon et al. considers swelling to be an integral part of the fluid in equilibrium [141]. Olsen et al. formulated a swelling model based on Biot's principle of effective stress by including the swelling directly in the effective stress [25, 141, 146]. Huyghe and Janssen extended the triphasic theory to a quadriphasic mixture theory which includes electric flux and potential gradients at finite deformations [147, 148]. A biphasic swelling model introduced by Wilson et al. [12] simplifies the triphasic and quadriphasic models by neglecting the electrolyte flux [143, 147] and assumes that the ion concentration remains always in equilibrium. 

After including the osmotic swelling in (1), the total stress of the material becomes

(15) $$\begin{array}{c} \sigma_{t}=\sigma_{nf}+\sigma_{\text{fibril}}-\Delta \pi I-\mu_{f}I, \end{array}$$

where Δ*π* is the osmotic pressure gradient and *μ*_*f*_ = *p* − Δ*π* is the chemical potential of fluid [12, 59, 147, 149]. The osmotic pressure gradient is caused by the difference in ion concentration of the cartilage and that of the surrounding fluid [12, 147, 149]. It is also referred to as the Donnan swelling pressure gradient. However, the mere osmotic swelling theory seems to underestimate the amount of swelling [150]. Two possible explanations for that are (1) the distinction of intra- and extrafibrillar water [150] and (2) chemical expansion [151]. The inclusion of chemical expansion further extends (15) to

(16) $$\begin{array}{c} \sigma_{t}=\sigma_{nf}+\sigma_{\text{fibril}}-\Delta \pi I-\mu_{f}I-T_{c}I, \end{array}$$

where *T*_*c*_ is the chemical expansion stress. This model has been applied earlier specifically for cartilage since its swelling properties due to the fixed charge density have a significant role for the deformation behavior of the tissue, especially under static loading. For the implementation of swelling properties, values of the fixed charge density can be obtained from experimental measurements [152, 153]. The fixed charge density (*c*_*F*_) affects both osmotic swelling and chemical expansion [142, 143]. In the biphasic swelling model, chemical expansion is only a function of *c*_*F*_ [142]. However, the inclusion of the chemical expansion term as proposed by Lai et al. [143] may not be appropriate, as it conflicts with the second law of thermodynamics. Huyghe et al. demonstrated that the chemical expansion stress part could produce free energy during closed loading cycles if the theory were true [144]. Nevertheless, osmotic swelling alone may not fully simulate the swelling behavior in cartilage [144]. However, when splitting the fluid phase into intra- and extrafibrillar portions [8, 150], implementation of chemical potential may not be needed.

As discussed earlier, the swelling of cartilage is resisted by the collagen network, inducing prestresses in the collagen fibrils. There are also other representations to the mechanical behavior of collagen fibrils than those presented above. Specifically, the nonlinear stress-strain tensile behavior of the collagen fibrils has been presented as follows:

(17) $$\begin{array}{c} P_{1}=E_{1}\left(e^{k_{1}\varepsilon_{f}}-1\right), \end{array}$$

(18) $$\begin{array}{c} P_{2}=E_{2}\left(e^{k_{2}\varepsilon_{e}}-1\right), \end{array}$$

(19) $$\begin{array}{c} P_{f}=P_{1}+P_{2}, \end{array}$$

where *P*_*f*_ is the first Piola-Kirchhoff fibril stress, *ε*_*f*_ is the total fibril strain, *ε*_*e*_ is the strain of the spring *μ*_1_, and *E*_1_, *E*_2_, *k*_1_, and *k*_2_ are constants [8, 64]. Again, the stress is computed only when the fibrils are in tension (i.e., *ε*_*f*_ > 0 and *ε*_*e*_ > 0; otherwise *P*_*f*_ = 0).

#### 2.1.4. Optional Solutions for Fibril Reinforced Material Simulation

The above-listed models present only the development of one branch of fibril reinforced models, applied in several studies. However, several other models used for understanding cartilage mechanics and etiology of OA have been proposed and applied as well. The principles of a few of these optional solutions are outlined below. 

Li et al. implemented spring elements to simulate fibril reinforcement [119]. This implementation combined two types of elements (springs and poroelastic matrix) overlaid on top of each other. Such implementation allowed for easy description of compression-tension nonlinearity in the cartilage, with collagen fibrils affecting cartilage total stress only in tension. In a quite similar manner, Shirazi and Shirazi-Adl simulated fibrillar matrix using directional membrane elements overlaid over poroelastic matrix [131]. The models provide similar results [131].

Olsen and Oloyede implemented an overlay model [140], which was designed to study articular cartilage fibril reinforcement in a swollen construct. They later formulated and applied the model for simulating transient responses [141]. In the model, cartilage solid was considered as a multilayer matrix, the layers of which all undergo the same deformation and contribute to the total stress. Using the overlay method, several different components could be applied in a single model, although the interactions between the overlays could not be implemented. 

Ateshian et al. [66] implemented the solid matrix of cartilage with a continuous fibril angle distribution including osmotic swelling behavior in the nonfibrillar matrix. They only simulated the equilibrium responses subsiding interstitial fluid flow and flow-independent viscoelasticity. The application of continuous fibril angle distribution was able to correctly simulate the compression-tension nonlinearities in tissue stress and deformation. Ateshian et al. found that most of the conducted predictions could not be obtained from models using only three orthogonal fibril bundles. However, they considered it possible that some of their simulated features may also be captured by discrete (vector-based) fibril models with more than three orthogonal fibril bundles.

Holzapfel and Gasser described a viscoelastic fibril reinforced structural model [135] applicable also for simulating mechanics of articular cartilage with three-dimensional stress and deformation. This model falls into the vector-based fibril reinforced models with two fiber directions embedded in an isotropic ground matrix describing the composite fibril reinforcement. Gasser et al. further developed the model to incorporate a structural parameter characterizing the dispersion of collagen fibrils [154], which was later applied for articular cartilage [63]. 

Pierce et al. [67] proposed a structurally motivated 3D fibril reinforced mixture model. Their model was able to simultaneously address the dependence of fibril reinforcement and permeability from the fibrillar structure but omitted flow-independent viscoelasticity. The model included a dispersion parameter representing the degree of anisotropy within the fibrillar matrix, which could potentially be adjusted to account for collagen fibrillation associated with tissue degeneration. The model was able to reproduce mechanical behavior of articular cartilage under a variety of loading scenarios and demonstrated good agreement with experimental results.

Quinn and Morel implemented an intuitive model for simulation of collagen network mechanics and interactions between the fibrillar and nonfibrillar matrices [155]. In their model, the fibril density and orientation can be utilized. Direction of the collagen fibrils is determined much like in the vector-based models presented above. This model separates the hydrated and dry portions of collagen and PGs, while also including “free” water and considering the structure of the fibrillar matrix. Although the solution presented by Quinn and Morel was restricted to equilibrium state, it could be extended to more general applications. 

#### 2.1.5. Controversies and Limitations Related to Tissue-Level Fibril Reinforced Models

Not all aspects of the biological cartilage tissue have been considered when attempting to simulate its mechanical behavior, and more specifically that of its fibrillar matrix. Limitations and challenges for implementation are caused by the different scales defining the mechanical appearance in the structure of the collagen fibrils and the coupling between chemistry, biology, and mechanical behavior. Aspects not often considered at tissue level include physical phenomena such as buckling of the fibrils [156–158], fibril-fibril interactions [159], fibril-PG interactions [14], or length and width of the collagen fibrils. The present fibril reinforced models also neglect some basic physiological phenomena such as fluid-pressurization-induced contact lubrication effects [160].

Buckling behavior of the collagen fibrils is rarely considered in fibril reinforced models of articular cartilage. However, it is likely that fibril buckling occurs in a confined space [161]. Schinagl et al. suggested that a microstructurally motivated model considering buckling of the collagen fibrils at a critical load might account for the experimentally observed softening of the material with increasing load, following an initially stiff response [157]. Bursac et al. modeled cartilage through a network of cables under pretension, representing collagen submitted to osmotic pressure. Under small deformations, the fibrils are in tension, which is reflected as high network stiffness. Instead, at larger deformations, the forces within the fibrils (represented as cables) become compressive, eventually leading to buckling with the result of reduced stiffness [161]. Hence, buckling of the fibrils in cartilage will likely contribute to the compressive stiffness of the tissue at large deformations.

Fibril-fibril interactions have not been considered in the tissue-level fibril reinforced models. Nevertheless, the characteristics of the fibrillar matrix are likely highly dependent on the micromechanical interactions of the collagen fibrils. For instance, cross-linking has been demonstrated to affect the properties of the collagen fibril network [136, 162]. Buehler demonstrated that high cross-link density is associated with high yield strength and stress and brittle failure, while low cross-link density is associated with more ductile behavior with strain softening following peak stress [162]. Chen and Broom suggested that breakdown in the interconnectivity of neighboring fibrils could lead to strongly aligned structure [163, 164]. Hence, any degenerative change in the fibrillar matrix could lead to rearrangement of fibrils along with varying degrees of fibrillar alignment. The loss of interconnectivity in the fibrillar network leaving the PGs intact could lead to reduction in PG entrapment accompanied by increased swelling tendency and decreased matrix stiffness [165]. Broom and Silyn-Roberts reported that matrix cohesion is a consequence of fibril-fibril linkage independent of PGs, and biomechanical models involving shear stress transfer between the fibrillar and nonfibrillar matrix may therefore be inappropriate [166].

Fibril network and nonfibrillar solid matrices have obvious mechanical interaction considering that the osmotic swelling due to fixed charges within the nonfibrillar matrix is balanced by the tensile properties of the fibrillar network [14]. Quinn and Morel highlighted the interactions between the collagen network and the PG gel [155]. They explained the collagen fibril prestress in free-swelling cartilage emerging naturally and without introduction of additional artificial model parameters. Prestressing the collagen fibrils can be simulated during free swelling.

According to the implementation of the nonfibrillar matrix as presented above (Section 2.1.1), the nonfibrillar matrix can resist load on its own without the fibrillar matrix confining it. However, this may not be correct [14, 139, 167]. Through the interaction between the PG gel and the fibrillar matrix, the nonfibrillar matrix is known to contribute to the compressive tissue stiffness [7, 70, 90, 168] and to greatly resist fluid flow [169, 170]. In the aforementioned mixture-type models, the fibrillar matrix could be theoretically removed from the mixture leaving only the fluid-saturated nonfibrillar matrix, which would still be able to resist load as a porous elastic or hyperelastic matrix. This controversy in the interaction of the fibrillar and nonfibrillar matrix is not often considered in the implementation of the fibril reinforced models. Instead, the nonfibrillar matrix is actually considered to include the effects of fibrillar matrix confinement of the PG gel. This is due to implementation of infinitesimally thin fibrils (and fibril bundles), using for instance, springs. Hence, the fibril reinforced models do not fully separate the collagen and PG contributions to cartilage mechanics, as the drained nonfibrillar matrix possesses a nonphysical Poisson's ratio giving the appearance that collagen fibrils support the nonfibrillar matrix even at zero tensile stress [14, 155]. Therefore the fibril reinforced models are still limited in their potential for estimation of cartilage mechanical properties from independently determined microstructural data and molecular physics [155].

Microstructural data such as fibril length and diameter are rarely considered in the implementation of tissue-level fibril reinforced models. However, it must be acknowledged that fibril length and diameter have impact for their mechanical performance affecting buckling and reorganization of the fibrils within cartilage tissue during external loading. Fibril diameter may relate directly to the mechanical properties of the tissue [171–173]. The length of the fibril also hypothetically affects the arrangement and reorientation of the fibrillar matrix during loading.

Past experimental measurements and theoretical predictions of the fluid pressurization of cartilage have demonstrated that the load support provided by interstitial fluid approaches the applied load immediately after loading, while recovering to zero over time during static loading [23, 24, 83, 85]. This excess fluid pressurization causes an issue with cartilage lubrication modifying the contact friction [174]. This aspect has been poorly incorporated into the presently applied (fibril reinforced) models, although a theory applied by Ateshian et al. [175] has been shown to agree well with experimental measurements [176]. 

### 2.2. Cell-Level Models and Cell-Tissue Interactions

**Figure 3:**
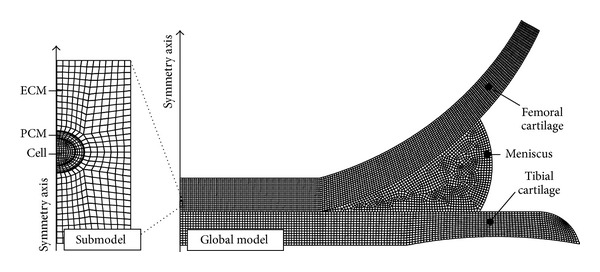
Multiscale modeling allows for simulation of macroscale effects on microscale. In this example, a macromodel (global model, on the right) was used to simulate knee joint function and the effects on a single chondrocyte in femoral cartilage were simulated in microscale using submodeling (on the left). The transient boundary conditions were driven by the global model.

Biphasic multiscale models were first introduced in early 2000 [177]. The goal of these models was to evaluate cell behavior in articular cartilage, cell-tissue interactions, and the effect of altered ECM and PCM properties for the deformation behavior of cells. The models have been applied to simulate cell-matrix interactions under steady state [132] and transient loading [59, 177, 178]. In the multiscale models, those parameters of the macroscopic tissue model that associate with the global loading (displacement, fluid pressure) serve as input parameters for the local submodel, including the chondrocyte and its local mechanical environment (Figure 3).

Recently, these multiscale models have incorporated the fibril reinforced constitutive laws of cartilage tissue presented in Section 2.1 [59, 132]. In addition to the ECM, the PCM around cells has been modeled. The PCM has also been modeled as fibril reinforced biphasic tissue and the fibrils have been implemented according to microscopic studies for tissue structure [132, 133]. This is because the loading-induced changes in the fibrillar patterns surrounding the chondrocytes likely transmit information from the matrix to cells causing metabolic changes in the cells [10]. Hence, incorporation of fibril reinforced models to study mechanical behavior of chondrocytes is appropriate. Chondrocytes have been typically considered using the same constitutive equations as the ECM and PCM, except that the fibrils have been neglected. See the typical material properties of the ECM, PCM, and cell typically implemented in multiscale models of cartilage (Table 2).

Fibril reinforced multiscale models of cartilage have emphasized the importance of cartilage structure, composition, and mechanical properties on cell responses [16, 59, 132]. Specifically, the arcade-like orientation of the collagen network in the ECM has been suggested to modulate the cell shape differently in different zones [59]. The fixed charge density of the ECM has been shown to increase cell aspect ratio especially in the superficial zone, while the fluid has been suggested to alter the transient behavior of cells [59]. The collagen fibrils in the PCM have been found to modulate the mechanical signals experienced by the cells and protect cells, while the fixed charge density in the PCM may significantly alter cell morphology and deformation behavior [58, 133].

### 2.3. Adaptation Models

Mechanobiology investigates the biological responses of the cell to physical loading. Hence, it involves understanding how the tissue adapts the geometry, composition, and structure to the mechanical loading it is exposed to. With respect to articular cartilage, it is believed that the specific anisotropic organization of the collagen fibers is a result of the mechanical stimulation in the joint during development and growth [179]. However, the rules by which the spatial organization of the collagen fibers is related to mechanical and chemical signals are not yet elucidated. With that in mind, algorithms that relate collagen remodeling and adaptation to stress and strain have been developed [179–181]. In the collagen remodeling algorithm, the collagen fibrils align along the preferred fibril directions that are situated between the positive principal strain directions [179, 180]. The simulation is conducted over time. After each step the collagen fibril orientation is updated and followed by an iteration where the new tissue strains are computed. This process is repeated until homeostasis is achieved. By combining the collagen remodeling algorithm with a fibril reinforced swelling model, Wilson et al. were able to predict the development of the typical Benninghoff-type collagen fiber orientation [179]. Nagel and Kelly applied the remodeling model to observe effects in collagen architecture of cartilage in the vicinity of transplanted tissue [181].

Mechanobiological models have frequently been used to investigate changes in tissue composition and tissue volumetric growth in bone remodeling, fracture healing, and growth plate development [182–185]. Thus far, these models have not been extended to articular cartilage although van Donkelaar and Wilson simulated the effects of PG and collagen synthesis on chondrocyte hypertrophy [186]. Such models may become useful in future studies addressing the mechanical influence on the progression of OA. In particular, combining mechanobiological adaptive models that evaluate tissue biosynthesis based on responses experienced by cells with imaging of the tissue geometry and composition may enable the development of patient-specific models to predict articular cartilage degradation and OA progression with time. 

## 3. Practical Considerations in Modeling Applications

At present fibril reinforced models of articular cartilage are inhomogeneous and anisotropic, requiring several components to describe the overall mechanical behavior. These complex models are required for advanced simulations in order to mimic cartilage behavior at the same time considering the computational limitations. With a great number of tissue components and material parameters, the models become prone to uncertainties caused by the assumptions, for example, in the interactions between the model components. These uncertainties need to be addressed and the model implementation and function should be verified. With a carefully designed and verified computational model, experimental tests can be simulated quickly, and cases which are not possible in an experimental testing setup can be investigated, for example, through parametric analysis. Although a model may be plausible in theory, a thorough validation is required before the model can be applied. Subsequently, computational fibril reinforced models can be applied for retrieving intrinsic properties of the tissue, which describe its mechanical behavior by combining experimental tests and model simulations [7, 17, 47, 64, 153, 187–190]. In the following, some general considerations, also necessary when applying fibril reinforced materials of articular cartilage, are reviewed and discussed.

### 3.1. Parametric Analysis and Design of Experiments (DOE)

Sensitivity of different model components for the mechanical response of cartilage can be evaluated using parametric analysis. In general, the evaluation can be important either for simulations and experimental designs or for optimizing model behavior. Fibril reinforced models can have several material parameters describing stress of the collagen fibril matrix in addition to stress of the remaining solid matrix and fluid flow. To successfully predict the experimental data, the model should have a limited number of unknown parameter values in order to optimize the inverse problem uniquely. With a low number of parameters in optimization, the optimization routine becomes more reliable and efficient. In addition, the optimized variables are required to have distinguishable effects on the simulated outcome. To limit the number of optimized parameters, it is important to know which model parameters most sensitively affect the outcome of the simulations and which have a minor effect on the outcome. This way, the critical parameters can be optimized, while the parameters with lower impact can be assumed and fixed during optimization. 

One common method of conducting parametric analysis is to vary one parameter at a time while keeping other parameters constant. This parametric analysis requires a basis, which is the set of reference parameter values which are then adjusted separately. The effect of these adjustments is then compared with the basis simulation. Several previous finite element analysis studies have used simple parametric analysis to either test the sensitivity of model parameters on the simulation outcome or to predict how the changes of model parameters would translate into observed changes in real-life physiology or the experiment outcome [20, 132, 191–193]. However, with such a simple method the interactions between the parameters cannot be captured or effectively predicted. Instead, a full factorial design could be used to test the effects of multiple parameters concurrently. In such case, the results of the parametric analysis are not affected by the basis simulation. Full factorial designs are suitable for studying systems with a low number of parameters and levels. They also provide more information on the interactions between the parameters. However, when the number of parameters and levels becomes higher, a full factorial design becomes unpractical due to the high number of required simulation runs.

One method for performing such multidimensional parametric analysis is the DOE approach using factorial analysis, which can also evaluate certain interactions between different model parameters [194]. A full factorial design is the most reliable and comes with the greatest computational cost. In a full factorial design, all unknown parameters are evaluated as a function of each other. In the case of the more advanced models, it is clear that all of the models have a large amount of model parameters, some of which have values that have been assumed. For such cases, the full factorial design may become too extensive, and a more efficient way to determine the most significant parameters for the simulation outcome is required. One such approach is the fractional factorial design, which can significantly reduce the number of factorial test runs [195, 196]. Fractional factorial design is therefore suitable when determining the model parameters that affect the simulation outcome the most in models with a large number of independent parameters. This may be suitable for some of the more complex fibril reinforced models of cartilage [6]. Different DOE methods should generally be used to reveal mechanisms that control the simulation outcome in advanced computational models [6, 183, 194, 197].

Although the applications of DOE for finite element analysis are numerous, it is still a quite new and seldom used technique in orthopaedic biomechanics [6, 183, 198–200]. The most popular design and method is the one by Taguchi et al. [201], which generally emphasizes the optimization of model performance by selection of the best values of the controllable parameters. We expect DOEs to gain more interest in the future also in finite element analysis of articular cartilage. The fibril reinforced models are becoming more complex and are being applied in more complex situations. Further, the need for optimized model design in material and geometry aspects becomes more important.

### 3.2. Considerations for Optimization

Curve fitting through optimization is an essential part of the application of fibril reinforced or any other biomechanical model, since material parameters describing model behavior can be derived to produce a simulation which corresponds with the experimental tests. These optimized material parameters then provide insight into the intrinsic tissue mechanical properties. Since there are various different constitutive models that can be used to simulate a similar outcome and agreement with certain experimental tests, it must be kept in mind that the optimized material parameters only describe the mechanical behavior of the tissue with the applied material model. Thus, the optimized material parameter values do not necessarily represent intrinsic tissue properties which can be generalized. Hence, the comparison of those material parameter values retrieved using fibril reinforced models should be conducted only between studies conducted using the same model. However, similarities in the overall principles between the different constitutive models may allow for comparison of the phenomena and trends observed between different studies and models.

As mentioned earlier, the modern models of articular cartilage include the description of many material parameters that cannot be measured directly. Due to the complexity of the fibril reinforced models, the fitting of the model to experimental data is often difficult through selection of model parameter values using the trial and error method. Therefore, optimization of parameter values against, for example, experimental data is conducted. During optimization, the model parameter values are often adjusted in a manner that will make the simulation result correspond with the experimental data as well as possible based on a defined objective function. In such a case, an objective function is minimized in case it represents an agreement error or maximized if the objective function represents the agreement between the experimental and simulated data [8, 12, 17–19, 47, 64, 65, 68, 98, 188, 189, 202–205]. Choice of the objective function is a major factor in determining the success of a fit. Most types of commercial simulation software, such as ABAQUS, COMSOL Multiphysics, provide a feasible platform to conduct modeling, and optimization can be done in conjunction with external software like MATLAB [189, 206] or Isight [207–209].

Prior to multidimensional optimization, it is suitable to run parametric analysis in order to choose only the sensitive model parameters for optimization. This will help to reach a unique solution. The optimized variables are required to have distinguishable effects on the simulated outcome for the solution to be unique. Generally, the lower the number of variables to be optimized, the more reliable and efficient the optimization routine. However, this may come with the cost of reduced agreement between the experimental and simulated data. The convergence of the optimization becomes quicker if the initial values are close to optimal [189]. Therefore, initial values should not be selected in random, but within reasonable range, for instance based on literature source, or screening with parametric analysis [188, 210]. Randomly selected or unrealistic initial values could potentially cause unnecessary problems in the convergence of the model.

Considering that the experimental data includes some error, for example, due to uncertainties related to experimental testing, an error in the optimization should be accepted within that error margin. Therefore, a satisfactory optimization could result in various sets of optimized values depending on the initial values of the model parameters prior to optimization. However, changing the initial values and conducting the optimization again should result in a quite similar set of optimized parameter values, provided that the parameters chosen for optimization were considered to affect the simulation outcome sensitively. Therefore, it may be beneficial to use several initial value sets to verify uniqueness of fit and to determine the range at which the parameter values lie within satisfactory objective function values [68, 189, 202, 211].

**Figure 4:**
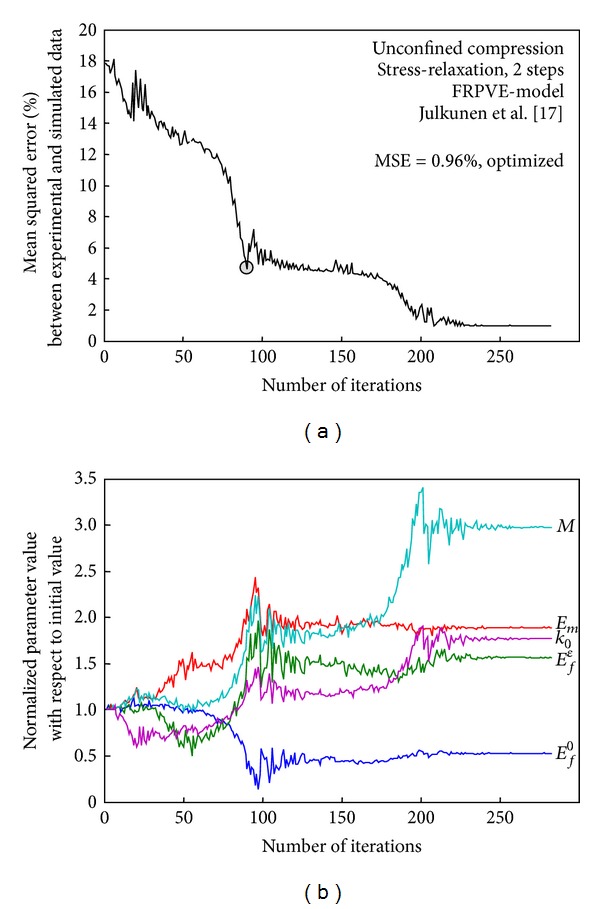
Convergence of an objective function (mean squared error, MSE) during the optimization of a 2-step stress-relaxation experiment [17]. (a) With initial parameters MSE was 17.73% and after optimization it was 0.96%. Five model parameters were optimized using a multidimensional minimization routine (the Nelder-Mead simplex method). One local minimum is indicated with a grey circle. (b) Normalized parameter values after each iteration with respect to the initial values. Less than 0.5% change between iterations in any of the optimized parameters was observed at the end of optimization, after the solution had converged.

Local minima are problems for the optimization (Figure 4), since they may result in fulfillment of some cutoff criteria for the optimization, providing one with a faulty set of model parameter values. A common practice in multidimensional optimization is to repeat the optimization routine in case of unsatisfactory objective function outcome (e.g., in a local minimum) by setting the optimum parameter value set as initial values for the following optimization (Figure 4). One must keep in mind that the chosen objective function has an effect on the optimized set of parameter values provided that complete agreement cannot be achieved [202]. Hence, the most appropriate objective function should be selected. Generally, when comparing experimental and simulated data, an error function comparing the two for absolute agreement is considered suitable. Instead, if a trend between the experimental and simulation data is optimized, it may be appropriate to use correlation between the two as an objective function. One could apply weight functions for each data point in use when assessing the success of a fit. The weight functions could be based on standard deviation of the experimental data at a specific data point to consider the uncertainty in accuracy of the experimental data.

### 3.3. Convergence Tests

**Figure 5:**
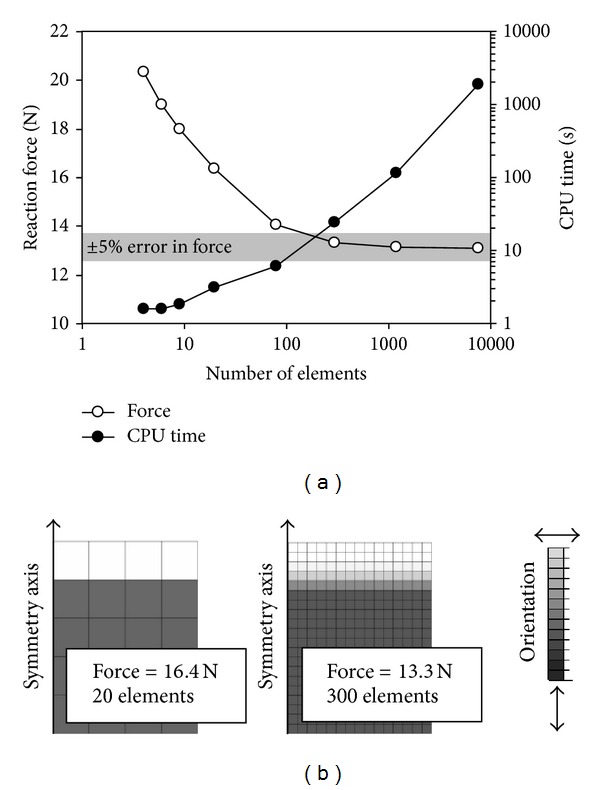
Convergence test for finite element mesh. As the number of elements is increased in an inhomogeneous finite element model, the simulation outcome begins to converge towards true value (i.e., case with infinite number of elements). In this case, the finite element mesh was homogenous and the number of elements was systematically increased to observe convergence in the simulated peak reaction force during unconfined compression experiment (a). To optimize the performance of the model, an appropriate amount of elements is required in the model to obtain reliable results (within 5% error from an excessive amount of elements). If an excessive number of elements are used, the computational cost (CPU time) increases, and the model performance suffers. In this demonstration, the model used was a fibril reinforced poroviscoelastic model [17, 18]. In the model, collagen fibrils are implemented with a Benninghoff-type arcade structure. Therefore, to observe the effect of the bending of the fibrils, it is essential to have a sufficient amount of elements. Two finite element meshes with the fibril orientations are shown (b).

The rather complex geometries required to model the joint behavior are prone to ineffective computing, provided that the mesh is not optimized. To optimize the mesh density and biases after determining the simulation geometry, a convergence test should be performed to determine the coarsest acceptable mesh, using the element type that can produce an outcome with acceptable error, compared with the ideal; that is, the converged outcome. The localized anisotropies within the material model, like in the fibril reinforced models may affect the convergence, and optimal mesh design (Figure 5). In the test, mesh density is increased and the simulated outcome is recorded. As the simulation sequence reaches critical mesh density, that is, denser mesh will not change the simulation outcome, the mesh with an acceptable error (e.g., <5%) can be used to simulate the overall outcome. An example of such test is presented in Figure 5. It is also noteworthy that the CPU cost increases with denser finite element mesh, which is an extremely important time-consumption issue when the sample-specific properties are solved through optimization.

When a model is intended to simulate localized phenomena in a more complex geometry (e.g., complete knee joint) or in multiscale, it is necessary to use a mesh dense enough to have the model converge also for the localized simulations. Therefore, a similar convergence should be run to confirm the accuracy of the simulations specifically at regions of interest.

### 3.4. Model Validation

All models should be validated for feasibly corresponding with reality. The overall goal for validation is to make the model applicable to address a problem of interest with sufficient accuracy and confidence. In model calibration/optimization, the model should be compared with a real set of experimental data, and acceptable limits of discrepancies between the simulated and experimental data should be obtained [8, 12, 18, 64, 65, 78, 186, 212]. Considering fibril reinforced material models of cartilage tissue, validation of the model may come with some unique challenges, such as how to validate the fibril reinforcement in a mixture model and the interactions between the fibrillar and nonfibrillar matrices. Validation against experimental data can be made with three different approaches: direct, indirect, and trend measurements [213]. 

Direct experimental measurements and comparison with simulation results serve as the most reliable validation of the model. Further confidence can be gained through the direct parametric analysis of experimental tests and then comparing the results with those predicted by the simulations. A good example of a direct validation of a model theory to predict internal fluid pressurization was conducted by Soltz and Ateshian [83, 85] and Olsen et al. [141]. However, direct validation is often difficult (e.g., pressure distribution in articular cartilage in a knee joint), since the simulated experiments cannot be replicated in controlled measurements with live subjects. For this reason, it may be required that indirect measurements are used instead. In such case, an experimentally measurable property can be compared with the simulation results, even though the measurable property is not needed. The measurable property must be selected so that it has a direct link with the model behavior being validated. However, indirect measurements validate the potential of a model to perform as required. Therefore, the importance of indirect validation should not be overestimated [213].

Sometimes the model is used for describing a certain kind of physiological behavior and it may not require direct or indirect validation for absolute agreement. In such case, trend measurements may be used to explain whether the model exhibits similar trends, for example, during parametric analysis. Trend measurements are especially important when validating the interactions between the model components (e.g., how the composition changes affect the measured mechanical response in articular cartilage). Although validation provides confidence in a model, it does not confirm that it agrees with reality. Each new model should be validated with the direct or indirect evidence of its function.

### 3.5. Experimental Tests

In the following subsection, we will discuss what factors should be taken into account when designing experimental mechanical tests at tissue and cell level in order to determine the material properties through optimization. We will also show how the models can be used to explain the experimentally observed phenomena.

#### 3.5.1. Tissue-Level Experiments

**Figure 6:**
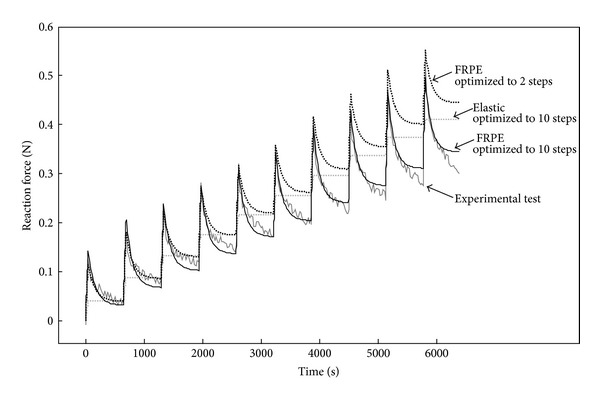
To demonstrate the role of experimental testing in optimization, we present a stress-relaxation experiment with 10 steps (2%-strain/step). We optimized 2 models (elastic and inhomogeneous fibril reinforced poroelastic, FRPE) to that test. The elastic model was fitted to all steps simultaneously by minimizing the mean squared error while the FRPE was fitted to 2 steps and 10 steps. The elastic model was unable to predict the data from the stress-relaxation curve, while the FRPE model agreed better with the experimental data. However, when the FRPE model parameters were optimized for 2-step data, the predicted data in the following 8 steps did not agree well with the experimental data. Instead, when the model was fitted to all 10 steps, a good agreement was achieved.

With regard to the fibril reinforced model of cartilage, unique material parameters can be obtained provided that the role of each model parameter for the measured output parameter has been taken into account in the experimental design. A simple way of characterizing three parameters of the fibril reinforced poroelastic model (fibril network modulus, nonfibrillar matrix modulus, and permeability) is to fit the model to a stress-relaxation experiment under unconfined or indentation geometry (Figure 6). The fibril network modulus controls primarily the peak forces, the nonfibrillar matrix modulus modulates the equilibrium forces, and the permeability affects the rate of relaxation. Thus, all three parameters have their own distinct effect on the measured stress-relaxation response.

When adding strain-dependent parameters of the collagen fibrils and permeability in the model ((6) and (8)), the number of model parameters increases and the nonlinearity has to be taken into account for example by increasing the numbers of steps in stress-relaxation experiments (Figure 6). Other option would be the inclusion of different experiments, for example, in other loading geometries [8, 12, 98]. This way the nonlinearity caused by the added parameters can be captured by the model. As an example, permeability and nonfibrillar matrix properties could be fitted to confined compression data, after which the fibril properties could be fitted on unconfined compression and/or indentation experiments. If further swelling parameters are added in the models (16), swelling tests could be performed simply by recording cartilage swelling and shrinking in media with different osmolarities [214, 215]. 

#### 3.5.2. Cell-Level Experiments

The cell-level biomechanical tests alone cannot be used for the optimization of model parameters. Instead, for model validation, experimental cell deformation behavior is compared with the corresponding computational analysis [216]. The model parameters of the ECM for such model validation should come from tissue-level experiments. Ideally, in order to have sample-specific material parameters also for the PCM and cell, microscopic-scale experimental tests should be employed. The mechanical properties of the PCM have been characterized with microaspiration technique [217]. The same technique can be used for measuring the mechanical properties of single cells [218]. Atomic force microscopy with nanoindentation can be used to characterize the small-scale biomechanical properties of the ECM and PCM, and it has also been used for the characterization of cell properties [219]. 

**Figure 7:**
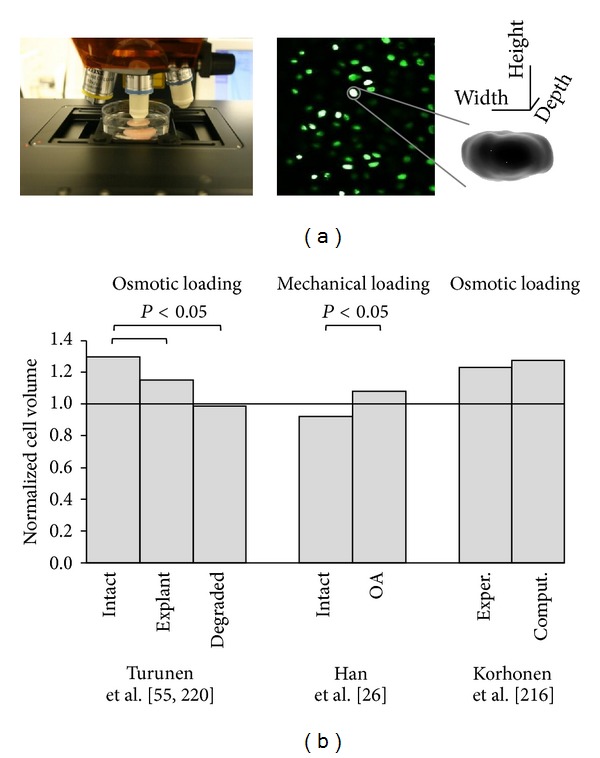
(a) The measurement setup of osmotic loading experiment of cells within intact cartilage tissue, a representative confocal microscopic image and a 3D presentation of a cell used for cell volume and morphology analysis. (b) Chondrocyte volume change in osmotically or mechanically loaded intact cartilage, cartilage explant, collagenase-degraded cartilage, and osteoarthritic cartilage (as a result of anterior cruciate transection). Experimentally determined cell volume change in osmotically challenged intact cartilage tissue is compared with a computational, microscale, fibril reinforced model (<1 ~ volume decrease, >1 ~ volume increase).

In order to test cell deformation within cartilage tissue, osmotic and mechanical loading of the tissue have been utilized simultaneously, concurrently recording the cells using confocal or dual-photon laser scanning microscopy [26, 55, 220]. In the osmotic loading experiments, diffusion of fluid in or out of the tissue is carried out by altering the osmotic environment of the immersion media. The hypotonic medium increases the osmotic pressure difference in and out of the tissue, creating tissue swelling. This causes swelling of cells as well. Cells have, however, the ability to recover their original state after the initial swelling [54]. This rapid recovery can be specifically seen using isolated cells. Some recent studies suggest that this recovery may be partly prevented or delayed by the ECM and/or PCM in the intact tissue [55], while chondrocytes in tissue explants and collagenase-degraded samples recovered back the original volume (Figure 7). Specific reasons for this phenomenon are, however, not known and computational modeling could provide an answer to such a problem.

Mechanical loading of articular cartilage, combined with simultaneous imaging of cells using confocal microscope, has been applied in unconfined compression and indentation geometries [26, 28]. Indentation combined with cell imaging can be used to characterize cell volume and morphology during compression. In this test, cartilage is intact, providing the physiological condition for cells. With this technique cell volumes were found to increase as a result of the mechanical loading of osteoarthritic rabbit cartilage, while the cells in normal joint cartilage were reduced in volume (Figure 7) [26, 27]. This technique, even using dual-photon microscope, can only reach some hundreds of microns into the tissue from the cartilage surface, limiting scanning to the superficial tissue layer. A technique in which chondrocytes are measured through the cut surface of tissue explants simultaneously with the compression of the cartilage surface can provide a way to characterize cell responses in the superficial, middle, and deep zones [28]. A limitation of this technique may be that cutting of the samples can damage the integrity of the samples and loosening of the collagen fibril tension around cells. This may eventually lead to different responses of cells. 

## 4. Visions for the Future

### 4.1. New Challenges

With the present cartilage models, supported by their further development, the new challenges for the future can be expected to advance from the generalization of theories and disease etiology more towards patient- and sample-specific simulations. At the same time, onset and progression of OA may be more specifically considered at a single-patient level as well as at a general level. For future applications, it is important to be able to predict how cartilage will react to changes in its mechanical environment. Important questions will arise, including: will cartilage be damaged and when, how will the damage progress, and can we predict the outflow of PGs? For future model development, it will be important to incorporate the production of the cartilage matrix [186] and incorporate tissue differentiation into cartilage models.

### 4.2. Combining *In Vivo* Imaging with Modeling

**Figure 8:**
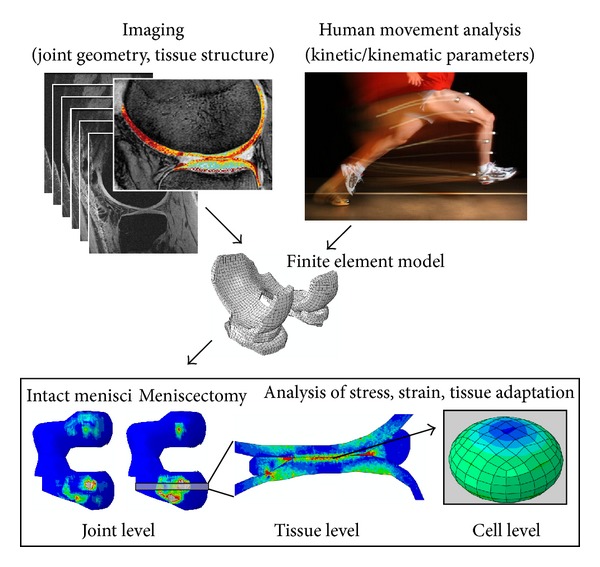
Subject-specific joint geometry can be imaged using, for example, MRI, from which using contrast agents, imaging protocols and image-analysis techniques structural and compositional details of articular cartilage can be measured and implemented into a finite element model. Using human movement analysis, the loading conditions can be determined realistically for individual subjects. Combining imaging, movement analysis, and finite element methods, realistic joint stresses and strains can be evaluated and effects on cells and matrix adaptation predicted using validated theories. Such analysis will aid in understanding and predicting advancement and onset of joint injuries and osteoarthritis.

In order to be able to evaluate the cell responses of a patient with a model, joint and tissue forces need to be obtained. In order to obtain the most realistic evaluation of those forces, two key points need to be implemented in the models in a patient-specific manner: geometry and material properties. The former can be done using for instance MRI (Figure 8). The latter must be taken from the literature because the mechanical properties cannot be measured from patients, even though some studies have characterized the relationships between the MRI parameters and biomechanical properties of cartilage [47, 221]. However, cartilage structure, directly related to the mechanical properties of the tissue, can be estimated from *in vivo* imaging techniques. T_2_ relaxation time mapping serves as a technique to quantify the arrangement of the collagenous network in cartilage [40, 222], and the effect of T_2_-based collagen orientation on stresses and strains of cartilage in a knee joint was modeled recently [62]. Diffusion tensor imaging may also be used to evaluate the collagenous structure of cartilage in undeformed and deformed state [63, 127, 129]. On the other hand, Gd-DTPA^2−^-enhanced T_1_ imaging (dGEMRIC) assesses the spatial changes in tissue PG content [223]. Contrast-enhanced cartilage tomography can be used to characterize the fixed charge density distribution of cartilage [224]. 

### 4.3. Toward Patient-Specific Estimation of Disease Progression and Treatment

The models presented in this paper and future adaptations of new fibril reinforced models could lead towards patient-specific estimation of the loading effects on cartilage degeneration in OA. By incorporating adaptive algorithms and phenomena behind tissue formation and degradation in the models, and combining these models with *in vivo* imaging and human movement analysis (Figure 8), it may be possible to develop computational models of entire joints. These models could be used, for example, to estimate the development of OA in patients with joint injuries (e.g., cartilage damage, meniscus tear, and ligament injury). 

Some computer simulation work has already been conducted with fibril reinforced material models of articular cartilage to understand and perhaps identify risk factors of OA development and progression [53, 115–117, 225]. In the future, modeling could be used for example for the evaluation of the effect of different clinical operations on the onset and progression of OA, in the first stage through stresses and strains of cartilage and in the later stages through adaptation models [179, 226, 227]. Modeling could also be used to evaluate the effect of different conservative strategies (for instance change in exercise) on the disease progression. Thus, the models could be used in the clinical decision making. Quantitative evaluation and prediction of the joint condition for the future would certainly aid in choosing the best treatment for the disease. When combined with potential repair materials, the computational models may have potential for optimizing and preparing growth protocols for those materials mimicking cartilage [228]. Furthermore, computational models should aid in the design of loading protocols for joint rehabilitation with degenerated cartilage, for example, when transplanting repair tissues that lack the mechanical requirements of the host tissue [229]. Recently Khoshgoftar et al. [230] investigated the potential of using a numerical model for stimulating formation of physiological collagen architecture in tissue-engineered cartilage. Such applications combined with the most modern models of tissue adaptation will certainly help to utilize mechanical loading regimes to benefit the optimizing of tissue-engineered cartilage [228]. 

## Figures and Tables

**Table 1:** Development of fibril-reinforced biphasic biomechanical models of articular cartilage.

Material model	Total stress	Primary material parameters	References
Fibril-reinforced poroelastic	**σ**_*t*_ = **σ**_*nf*_ + **σ**_*f*_ − *p * **I**	*E*_*f*_, *E*_*nf*_, *ν*_*nf*_, *k*	[7, 119, 131]
Fibril-reinforced poroviscoelastic	**σ**_*t*_ = **σ**_*nf*_ + **σ**_*f*_ − *p * **I**	*E*_*f*_^0^, *E*_*f*_^*ε*^, *η*, *E*_*nf*_, *ν*_*nf*_, *k*	[17, 18]
Fibril-reinforced poroviscoelastic swelling	**σ**_*t*_ = **σ**_*nf*_ + **σ**_*f*_ − Δ*π * **I** − *μ*_*f*_ **I**	*E*_*f*_^0^, *E*_*f*_^*ε*^, *η*, *E*_*nf*_, *ν*_*nf*_, *k*, *c*_*F*_	[12]

**σ**_*t*_: total stress; **σ**_*nf*_: stress of the nonfibrillar matrix; **σ**_*f*_: stress of the fibrillar matrix; *p*: fluid pressure; **I**: unit tensor; Δ*π*: osmotic pressure gradient; *μ*_*f*_: chemical potential of fluid.

**Table 2:** Typical material parameters implemented for the cell, pericellular matrix (PCM), and extracellular matrix (ECM) in the fibril-reinforced biphasic multiscale models [16, 59, 132, 133].

Material parameter	Chondrocyte	PCM	ECM
*E*_*f*_^0^ (MPa)	—	0.2	2.7
*E*_*f*_^*ε*^ (MPa)	—	34	340
*η* (MPa s)	—	947	947
*E*_*nf*_ (MPa)	0.002	0.038	0.5
*k* (×10^−15^ m^4^/Ns)	1000	0.1	1
*ν*_*nf*_	0.3	0.15	0.15
*c*_*F*_ (mEq/mL)	0.08	0.26	0.20
